# Parental Nitrogen Transfer and Apparent Absence of N_2_ Fixation during Colony Foundation in *Coptotermes formosanus* Shiraki

**DOI:** 10.3390/insects9020037

**Published:** 2018-03-26

**Authors:** Aaron Mullins, Nan-Yao Su

**Affiliations:** Department of Entomology and Nematology, Fort Lauderdale Research and Education Center, Institute of Food and Agricultural Sciences, University of Florida, Fort Lauderdale, FL 33314, USA; nysu@ufl.edu

**Keywords:** brood care, parental investment, dinitrogen

## Abstract

Colony foundation and early growth is a critical period in the life-cycle of a termite colony, as the initial family unit is resource limited. One such resource is nitrogen, which is essential for initial colony growth. This study examined the whole-colony nitrogen inventory during foundation and early growth of *Coptotermes formosanus* Shiraki colonies. It was hypothesized that termite colonies would go through an initial period of parental investment, representing a transfer of nitrogen to the first brood, and that once a functional worker caste was present, further provisioning in the form of intrinsic N_2_ fixation would occur. Our results showed that, when in nitrogen-poor rearing conditions, the king and queen initially transferred half of their nitrogen reserves to their first brood. However, the total nitrogen content in colonies did not increase over a 12 month period, despite the presence of functional workers. Furthermore, colonies did not increase their biomass beyond the initial parental investment. Together, these results imply that nitrogen acquisition in incipient *C. formosanus* colonies relies on environmental or dietary sources, rather than the putative fixation through symbiotic diazotrophs.

## 1. Introduction

Biologically available fixed nitrogen is a critical limiting factor in most terrestrial ecosystems. Conservation and procurement of nitrogenous resources is particularly an issue for wood-feeding termites, such as *Coptotermes formosanus* Shiraki, whose diet is extremely high in carbohydrates primarily as cellulose, but markedly low in protein [1]. As a group, the termites have many characteristics that are commonly referenced as adaptations for nitrogen conservation: a thin cuticle [2], the evolution of eusociality [3], storage excretion of uric acid [4], and cannibalism [5]. Although termites are highly specialized at nitrogen conservation, nitrogen procurement must take place in order to account for colony growth.

In terms of nitrogen procurement, wood-feeding termites are not necessarily limited to the scant protein present in sound wood. Wood rot fungi in decomposed wood may sequester nitrogen, and many termite species have been documented to selectively forage for nitrogen-rich resources within their feeding repertoire [6]. Termites have also developed a symbiotic means of nitrogen procurement, the intrinsic fixation of atmospheric N_2_ by diazotrophic bacteria residing in the hindgut paunch [7,8,9,10]. While dietary nitrogen is sought after, wood feeding termites supplement their nitrogen procurement with intrinsic N_2_ fixation [11]. Many species of diazotroph have been recorded residing in the hindgut of termites [12]. These associations can be quite intricate, involving obligate bacterial symbionts residing in obligate protist symbionts of termites [13].

**Figure 1:**
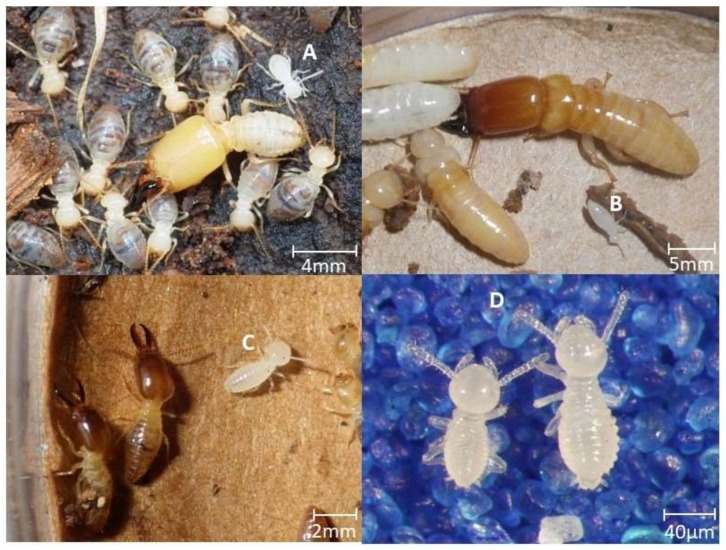
Representatives of three termite families, showing first or second instar larvae with transparent bodies and no evidence of food in the form of wood in the gut. (**A**) *Neocapritermes* sp. (Termitidae), (**B**) *Neotermes* sp. (Kalotermitidae), (**C**) *Microcerotermes* sp. (Termitidae), (**D**) *Coptotermes*. (**A**–**C**) photo credit: Rudolf Scheffrahn, University of Florida FL-REC.

At colony foundation, nitrogen requirements are high in order to obtain initial colony growth. Nitrogen fixation is an unlikely aid at this time, because the first two termite larval instars are transparent, and have an incomplete gut with no discernable food (wood) present (Figure 1) [14]. In addition, the king and queen have a very low titer of gut fauna compared to workers [15,16].

Termite colonies are founded by male and female alates, which have flown from a parent colony following a dispersal flight [17,18]. These alates pair off, mate, lay eggs, and ultimately care for offspring until the emergence of fully functional workers (at least third instar larvae) [2]. This period is unique in the life-cycle of a termite colony, as they may have extremely limited access to resources [17].

During colony foundation, brood care is strictly biparental, and provided by the king and queen, owing to the lack of a worker caste. However, this parental care quickly transfers to alloparental once a functional worker caste is present [19]. The term “parental investment” has been defined by Trivers [20] as “any investment by the parent in an individual offspring that increases the offspring’s chance of surviving (and hence reproductive success) at the cost of the parent’s ability to invest in other offspring”. In termites, the initial biparental investment is provided in the form of nest construction, defense, grooming, and nutritional provisioning of the first clutch of workers prior to their nutritional independence [3]. Although nitrogen requirements at colony foundation must be high in order to account for colony growth, very few studies have been done investigating the initial parental investment of nitrogen in primary reproductives [11], and no comprehensive studies have been done involving a complete nitrogen inventory of all castes during colony foundation of *C. formosanus*.

In this study, we hypothesize that during colony foundation, nitrogenous reserves in reproductives of *C. formosanus* will decrease until the presence of workers of at least the third larval instar (who have a complete gut), representing the parental nitrogen investment into progeny. Further, we expect that the total nitrogen content of the colony would begin to increase only once functional workers are present, and can provide procurement in the form of dietary nitrogen from consumed wood, and atmospheric N_2_ fixation.

In order to test this hypothesis, we initiated colonies of *C. formosanus* in a nitrogen-limited environment of wood and inorganic sand. A full nitrogen inventory of all castes was conducted through their first year of growth. The resulting data should allow for quantification of parental investment of stored nitrogen, and the subsequent contribution of the worker caste in terms of procurement. If an increase in nitrogen occurs during colony growth, and dietary nitrogen during that period can be accounted for, then any net increase can be attributed to newly procured nitrogen through fixation of atmospheric N_2_.

## 2. Materials and Methods

A single spruce wood *Pinus* sp. board was selected (2.4 m × 5.1 cm × 10.2 cm), which exhibited an even and uniform woodgrain throughout. The board was cut into cubes measuring 2 cm^3^. Cubes containing knots or other irregularities were discarded. The cubes were then oven dried at 90 °C for three days in individual plastic snap-cap vials (4 cm in diameter by 6 cm high) (US Plastics, Lima, OH, USA). At the end of the drying period, the individual containers were closed with airtight lids, and transferred to an airtight desiccator for cooling. After cooling for two hours in the desiccator, the containers were individually weighed, the wood block was removed, and the container was re-weighed. The difference in weight resulted in absolute dry weights for each of the 2 cm^3^ wood cubes. Three of these cubes were chosen at random, and samples (minimum 1.5 mg) were carved off and subjected to CHN elemental analysis in a Perkin-Elmer^®^ Series II CHNS/O Analyzer Model 2400 (PerkinElmer, Waltham, MA, USA). Ethylenediaminetetraacetic acid was used as a reference standard, and tomato leaf standards were used as a sampling control.

Individual snap-cap vials used in the drying process were prepared for incipient colony introduction and growth. Each container was filled with 25 mL of ethanol washed sand (Quickrete, Atlanta GA), rinsed with deionized water, and placed in a drying oven at 100 °C for 24 h. The 2 cm^3^ wood cube was placed in the container, and the block and sand were moistened with 12.5 mL deionized water. A total of 300 containers were prepared. The containers were covered with airtight lids with pinholes in the top for air circulation. Water was occasionally added to the containers to compensate for water loss through evaporation.

Alates of *C. formosanus* were collected during nuptial flights that occurred on the evenings of 21 and 25 May 2014 in Broward County, FL, USA. These alates were collected using a light trap [18], brought back to the lab, and after dealation, a single male and female were placed in each container with wood and sand. Ten pairs (five and five from the two evenings, consecutively) were frozen for later drying and nitrogen analysis (day zero). The containers with nuptial pairs were then stored at 27.2 °C, 34% RH. As needed, the containers were opened, and water was added to maintain moisture content.

For one year, ten containers with successfully established colonies were selected and destructively sampled every two months of development. Successful colonies were defined as colonies which had two surviving reproductives and their brood. Post-consumption wood blocks were cleaned, dried, and re-weighed according to the methods described above. The termites were removed and separated by caste: reproductives, eggs, first instar larvae, second instar larvae, all instars from the third instar larvae and higher (all combined as functional workers), and soldiers. Termites were dried at 60 °C for 24 h prior to being weighed. After weights were recorded, the termites were ground with a Pyrex^®^ pestle tissue grinder (Corning, Corning, NY, USA), and a sample aliquot was taken to measure total nitrogen for each stage of development in each incipient colony. Elemental analysis was conducted in the CHN analyzer using the same protocol stated above. In the case of eggs, first and second instar larvae, there was not enough biomass to measure each colony individually, so they were pooled across colony samples to provide one sample for each sampling date. Total nitrogen in each colony was calculated from the weight and the pooled total nitrogen value.

Net total nitrogen for colonies was calculated by summing all nitrogen present by weight in each colony biomass and subtracting the estimated dietary nitrogen (nitrogen content of the wood which had been consumed). In order to test for colony growth, the number of individuals of all castes in each colony at each sampling time were compared using a one-way ANOVA at α = 0.5 (Factor: time, variable: Total N, Net N). In order to test for significance in nitrogen content and biomass of colonies between different sampling periods, a one-way ANOVA at α = 0.5 (Factor: time, variable: Total N, Net N) was conducted for total nitrogen present in the reproductive caste, as well as for total biomass at each sampling period. Normality assumptions for all statistics were confirmed with normal quantile plots of the residuals. Tukey’s HSD (honest significant difference) was used as a post hoc test to separate means into categories of significant differences.

## 3. Results

**Figure 2:**
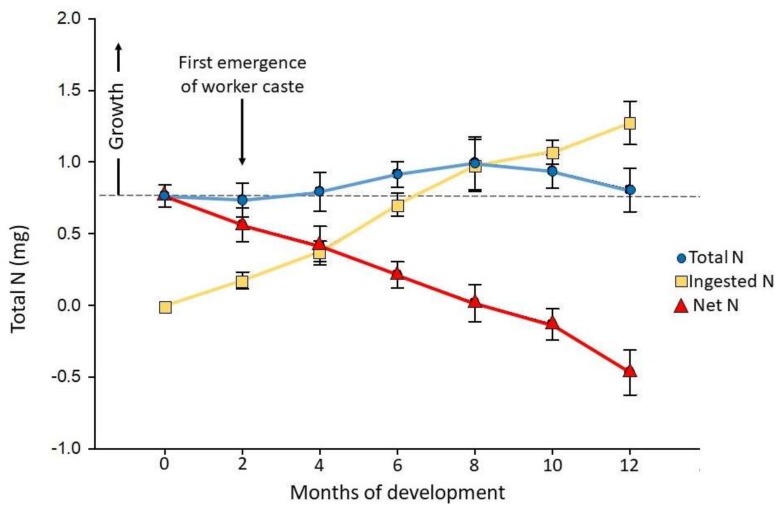
Nitrogen calculated in incipient colonies of *Coptotermes formosanus* during the first year of development. All data points (circles, triangles, and squares) represent means ± SE of 10 colonies. Total nitrogen (circles) represents the total mg of nitrogen present in all termite biomass of each colony. Ingested nitrogen (squares) represents the cumulative dietary nitrogen mg ingested from the wood diet provided to the colonies. Net nitrogen (triangles) represents the dietary nitrogen mg consumed from wood subtracted from the total nitrogen mg in termite biomass for each colony.

The maximum potential intake of nitrogen from the wood diet used as a food source measured, on average, 0.049% N (±0.005) by weight. Numbers of individuals and biomass of each caste during the growth period of one year is presented in Table 1. Growth in terms of biomass tended to stop after the first six to eight months of development. Growth in terms of number of individuals stopped after four months of development, and colony sizes remained constant from months 4 to 12. Eggs and brood were present at two and four months, and a smaller second brood was present at eight and ten months. Total and net nitrogen present in termite colony biomass, along with nitrogen acquired from the wood consumption is presented in Figure 2. As the colonies grew, the total nitrogen remained relatively constant; however, net nitrogen (the total nitrogen in termite biomass minus nitrogen missing in the wood) decreased significantly during the test period. During the first year of colony growth, net nitrogen decreased, indicating that the colony rearing unit was only able to maintain nitrogen levels through the input of dietary nitrogen. Functional workers were present starting at month four, but no uptake of net nitrogen was observed.

**Figure 3:**
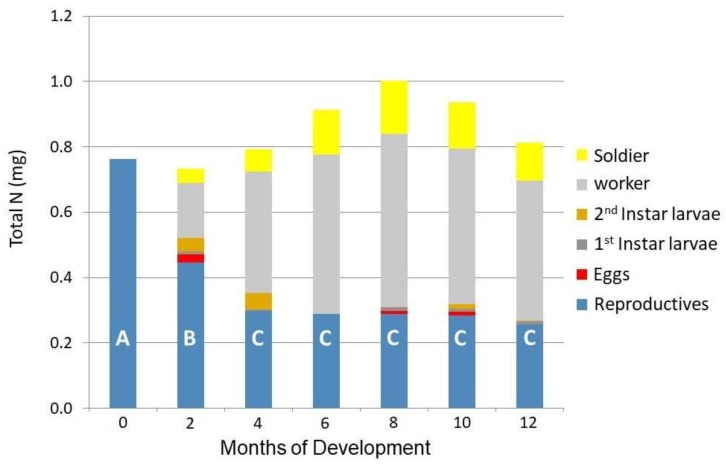
Distribution and transfer of total nitrogen by age and instar in incipient colonies of *Coptotermes formosanus*. Letters (A, B, C) in columns represent significant differences in nitrogen present in the reproductive caste (ANOVA α = 0.05, *p* < 0.001, post hoc Tukey’s HSD).

Figure 3 shows the total nitrogen present in each age and caste of the developing colonies. From zero to four months there was a marked decrease in total nitrogen of the reproductives. Nitrogen was primarily transferred to the first individuals to emerge from the first laid eggs, and by the fourth month of development, workers represented approximately half of all nitrogen present in the colony. No change in whole colony nitrogen content was observed during the first year of colony growth in nitrogen-poor conditions. 

## 4. Discussion

During the initial four months of development, the reproductive pairs lost half of the total nitrogen present in their biomass. However, after four months, their total nitrogen remained constant. Thus, the first four months of the life of the incipient colony represents the only biparental investment period, with roughly half of their initial nitrogen reserves transferred to their first brood (Figure 3). The nitrogen transferred was likely mobilized from uric acid reserves in the fat body [4], and from storage hexomerins, and post-flight wing muscle proteins that were atrophied after wings were removed [21]. Nitrogen tended to transfer in the largest part to first instar workers. It is unclear in what form the stored parental nitrogen was transferred to the offspring. Traditional trophalaxis of gut contents containing the termite’s diet of wood is unlikely, due to the very clear nature and lack of visible food in the gut of first and second instar larvae (Figure 1). A brood food based on pharyngeal gland secretions has been proposed in several higher termites [14,15,22]. Nalepa [23] documented such a brood food in the wood-feeding, nitrogen deprived *Cryptocercus punctulatus* Scudder cockroaches, the sister lineage to termites. During the development of early instar brood, *Cr. punctulatus* adults lose body weight and stored protein [3]. The current research provides a quantification of the nitrogen investment at the colony foundation level in *C. formosanus*.

During the course of this experiment, we observed a consistent reduction in net nitrogen present in colony units. The question remains as to why the colonies failed to grow even after four months of development when first instar workers were numerous. These first instar workers had functional guts with food that could be observed through the cuticle. We had hypothesized that once first instar workers were present, their contribution in the form of intrinsic N_2_ fixation would have increased the net nitrogen to well above the initial investment on the part of the alates. This was a reasonable prediction, as initial logistic growth usually occurs within the first year [20,24]. However, the colony rearing units were only able to maintain their nitrogen content (and biomass) through dietary intake from the nitrogen-poor wood (Figure 2).

Prior studies measuring nitrogen fixation rates of this species have returned results that are highly variable, with time required to double their nitrogen content through fixation ranging from one to 375 years [9,25]. In our study, the absence of net colony growth in both nitrogen and biomass may suggest that in *C. formosanus*, atmospheric N_2_ fixation does not occur in levels high enough to allow for colony growth in the first year of development. The steady decrease in net nitrogen observed was likely due to unavoidable and unmeasurable loss in the form of excretion, which was not accounted for in this study. This was due in part to the fact that subterranean termites excrete only trace amounts of uric acid [4]. In addition, feces were too difficult to separate from the sand substrate with any reliable recovery.

The fact that colonies maintained nitrogen content through their diet, but failed to grow in numbers after four months, provides evidence that optimal growth may only occur if provided with an alternative, nitrogen-rich food source. It has been observed that founding reproductives of *Zootermopsis nevadensis* Hagen were more likely to successfully produce offspring when their diet was artificially supplemented with uric acid [26]. The same author found a positive correlation between the cambium nitrogen content of a host log and the density of alate production from *Z. nevadensis* colonies living there [27]. However, other studies into the effects of dietary nitrogen on reproductive castes have not all found a positive relationship with nitrogen supplementation and fecundity [28]. In *C. formosanus*, optimal growth was observed in large lab colonies which were provided with nitrogen-rich organic soil, in addition to the same spruce wood and sand used in this study [19]. 

This study confirmed the initial parental investment of nitrogen transferring to the first brood during colony foundation. However, once depleted, atmospheric N_2_ provisioning by first generation workers was insufficient to allow for colony growth within the first year. Thus, our study challenges the assumption that atmospheric N_2_ fixation is an important source of nitrogen for colony growth in all termite species, at least in the first year [11]. It should be noted that at the end of the study, colonies did not seem to be moribund, and the study likely could have continued further. One limitation to our experimental design is that the diet provided to the termites had to be uniform and simple, in order to properly quantify nitrogen content and uptake. It has been reported that supplementing the diet of certain termites can result in lower N**_2_** fixation rates [9,29]. However, other studies have found no such relationship [30]. In nature, termites are known to selectively feed on certain nitrogen-rich parts of available food in their environment [6,26,27]. Unlike drywood termites in the family Kalotermitidae, *C. formosanus* is a subterranean termite species with access to soil. In our own experience, termite colonies given access to soil tend to be healthier, and this phenomenon has been previously reported [31]. It is possible that in order to grow, this species relies on alternative nitrogen resources present in their environment, rather than metabolically expensive intrinsic N_2_ fixation in their gut. Further investigations into the dietary nitrogen needs of a growing *C. formosanus* colony will be conducted in the future.

## 5. Conclusions

A biparental investment period of four months after colony foundation was determined for this species of termite. During this time, roughly half of all the nitrogen present in the founding reproductive pair was transferred to the offspring. Colonies failed to grow in numbers of individuals after four months of development. Nitrogen present in whole colonies also failed to increase during the entire study, even after the first few months, once functional workers were present. This provides evidence that nitrogen procurement in the form of intrinsic N_2_ fixation does not occur in this species during colony foundation and early development. We speculate further that dietary nitrogen may be an important resource for *C. formosanus* colonies during this period of development.

## Figures and Tables

**Table 1:** Average number of individuals ± SD (in parenthesis) and mass of each caste ± SD in 10 colonies of *Coptotermes formosanus* during one year of growth. Total biomass is a sum of the mass of all castes. Letters (^A^, ^B^, ^C^) indicate significant differences in total number of individuals in each colony during the growth period (ANOVA α = 0.05, *p* < 0.001, post hoc Tukey’s HSD). Letters (^a^, ^b^, ^c^) indicate significant differences in biomass during the growth period (ANOVA α = 0.05, *p* < 0.001, post hoc Tukey’s HSD).

Month of Development	Reproductives	1st Instar Larvae	2nd Instar Larvae	Worker	Soldier/Presoldier	Eggs	Colony Totals
0	(2 ± 0) 8.53 ± 0.79 mg	N/A	N/A	N/A	N/A	N/A	(2 ± 0) ^A^ 8.53 ± 0.79 mg ^a,b^
2	(2 ± 0) 3.89 ± 0.71 mg	(2.4 ± 1.71) 0.11 ± 0.17 mg	(3.2 ± 2.00) 0.39 ± 0.40 mg	(10.0 ± 4.27) 1.79 ± 0.87 mg	(1.7 ± 1.25) 0.42 ± 0.43 mg	(8.8 ± 7.89) 0.30 ± 0.28 mg	(21.3 ± 7.67) ^A^ 6.90 ± 1.88 mg ^b^
4	(2 ± 0) 2.79 ± 0.38 mg	(0.4 ± 0.70) 0.04 ± 0.09 mg	(4.0 ± 5.12) 0.49 ± 0.72 mg	(22.0 ± 9.01) 4.35 ± 1.63 mg	(2.3 ± 1.06) 0.96 ± 0.60 mg	(0.0 ± 0.0) 0.00 ± 0.00 mg	(32.7 ± 9.13) ^C^ 8.63 ± 1.78 mg ^a,b^
6	(2 ± 0) 2.71 ± 0.23 mg	(0.2 ± 0.42) 0.00 ± 0.00 mg	(0.1 ± 0.32) 0.01 ± 0.03 mg	(25.5 ± 2.95) 5.67 ± 0.74 mg	(3.6 ± 0.84) 1.94 ± 0.40 mg	(0.0 ± 0.0) 0.00 ± 0.00 mg	(33.4 ± 3.20) ^C^ 10.33 ± 1.06 mg ^b,c^
8	(2 ± 0) 2.71 ± 0.31 mg	(0.5 ± 0.85) 0.01 ± 0.032 mg	(0.2 ± 0.42) 0.01 ± 0.03 mg	(27.1 ± 5.95) 6.17 ± 1.52 mg	(4.6 ± 1.65) 2.28 ± 0.74 mg	(3.8 ± 3.99) 0.00 ± 0.00 mg	(36.4 ± 7.97) ^C^ 11.31 ± 2.26 mg ^c^
10	(2 ± 0) 2.68 ± 0.20 mg	(1.6 ± 1.71) 0.09 ± 0.13 mg	(2.2 ± 1.23) 0.00 ± 0.00 mg	(22.8 ± 3.01) 5.52 ± 0.97 mg	(3.9 ± 0.99) 2.01 ± 0.57 mg	(3.6 ± 4.67) 0.00 ± 0.00 mg	(34.5 ± 2.84) ^C^ 10.57 ± 1.38 mg ^b^^,^^c^
12	(2 ± 0) 2.41 ± 0.31 mg	(0.5 ± 0.71) 0.01 ± 0.03 mg	(0.5 ± 0.71) 0.02 ± 0.04 mg	(21.9 ± 4.9) 4.99 ± 1.21 mg	(3.7 ± 0.95) 1.64 ± 0.50 mg	(0.2 ± 0.42) 0.00 ± 0.00 mg	(30.6 ± 6.22) ^C^ 9.07 ± 1.81 mg ^a,b^
